# Psoriasis and Diabetes, a Dangerous Association: Evaluation of Insulin Resistance, Lipid Abnormalities, and Cardiovascular Risk Biomarkers

**DOI:** 10.3389/fmed.2021.605691

**Published:** 2021-03-23

**Authors:** Valeria Brazzelli, Pamela Maffioli, Vittorio Bolcato, Christian Ciolfi, Angela D'Angelo, Carmine Tinelli, Giuseppe Derosa

**Affiliations:** ^1^Institute of Dermatology, Fondazione Istituto di Ricovero e Cura a Carattere Scientifico Policlinico San Matteo, University of Pavia, Pavia, Italy; ^2^Department of Internal Medicine and Therapeutics, Centre of Diabetes and Metabolic Diseases, University of Pavia and Fondazione Istituto di Ricovero e Cura a Carattere Scientifico Policlinico San Matteo, Pavia, Italy; ^3^Centre for Prevention, Surveillance, Diagnosis and Treatment of Rare Diseases, Fondazione Istituto di Ricovero e Cura a Carattere Scientifico Policlinico San Matteo, Pavia, Italy; ^4^Department of Public Health, Experimental and Forensic Medicine, Forensic Science Section, University of Pavia, Pavia, Italy; ^5^Laboratory of Molecular Medicine, University of Pavia, Pavia, Italy; ^6^Clinical Epidemiology and Biometric Unit, Fondazione Istituto di Ricovero e Cura a Carattere Scientifico Policlinico San Matteo, Pavia, Italy

**Keywords:** psoriasis, type 2 diabetes mellitus, insulin-resistance, endothelial parameters, obesity

## Abstract

**Aims:** Psoriasis is an immune-mediated dermatosis with cardio-metabolic comorbidities. The aim of this study was to assess insulin-resistance, lipid abnormalities, and cardiovascular risk biomarkers in psoriatic patients with or without type 2 diabetes mellitus (T2DM).

**Methods and materials:** We enrolled 425 patients: 86 psoriatics, 69 psoriatics with T2DM, 120 T2DM patients, and 150 healthy subjects. We measured the Psoriasis Area and Severity Index (PASI), body mass index (BMI), insulin-resistance parameters [glycosylated hemoglobin (HbA_1c_), fasting plasma glucose (FPG), fasting plasma insulin (FPI), and with homeostasis model assessment index (HOMA index)], lipidic panel, plasminogen activator inhibitor-1 (PAI-1), homocysteine, soluble adhesion molecules, matrix metalloproteinase, and adipocytokines.

**Results:** FPG, HbA_1c_, and HOMA-IR were higher in diabetics with psoriasis (*p* < 0.0001) than in psoriatics. FPI levels were higher in diabetics with psoriasis than in diabetics and psoriatics (*p* < 0.0001), and higher in psoriatics than controls (*p* < 0.0001). Psoriatics and diabetics with psoriasis showed higher triglyceride and LDL-C levels (*p* < 0.0001) than diabetics. Homocysteine was higher in psoriatics and diabetics with psoriasis (*p* < 0.0001) than in diabetics. PAI-1 was higher in diabetics with psoriasis than diabetics (*p* < 0.01). sICAM-1 and sVCAM-1 were higher in diabetics with psoriasis than diabetics (*p* < 0.001 and *p* < 0.01) and psoriatics (*p* < 0.001 and *p* < 0.0001). Visfatin and resistin were lower in psoriatics (*p* < 0.0001) and in diabetics with psoriasis (*p* < 0.001 and *p* < 0.0001, respectively) than diabetics.

**Conclusions:** A limitation of this study is that there is a significant difference in mean age between controls and other study groups: the lack of matching between case and control groups may interfere with the external validity of the study findings. Despite this, the study highlights a pathogenetic link between psoriasis, considered a pre-diabetic condition, and diabetes. Insulin-resistance seems to be the keystone of psoriasis comorbidities. Psoriasis reinforces diabetes, causing a greater cardiometabolic risk.

## Introduction

Psoriasis (PsO) is an immune-mediated inflammatory disease (IMID) affecting the skin and characterized by keratinocyte hyperproliferation and T-lymphocyte inflammation, with a world prevalence of 2–4% (1). The pro-inflammatory molecules released during chronic inflammation may lead to one or more co-occurring disorders, such as atherogenesis and atherosclerosis, insulin-resistance, hypertension, obesity, dyslipidemia, metabolic syndrome, and type 2 diabetes mellitus (T2DM) (2–4). In literature, patients with psoriasis have an increased risk of new-onset of T2DM (5). Several epidemiological studies reported that the average prevalence of T2DM in psoriatic cohorts was 11.6% (6). However, other studies that examined the prevalence of T2DM in psoriatic patients compared to controls showed heterogeneous data that do not allow to make a realistic estimate, but sustain an increased prevalence of T2DM in psoriatic cohorts compared to healthy subjects, especially for late onset psoriasis (6). Moreover, a no correlation was observed between T2DM prevalence and psoriasis severity (6).

These two diseases share a common genetic etiology (7, 8) and several pathophysiological pathways (9) including those of inflammatory cytokines (10), adipocytokines (10), glucagon-like peptide-1 (GLP-1) receptor, and incretin (11, 12).

It was hypothesized that systemic inflammation in psoriasis causes insulin-resistance, an independent T2DM risk factor (13, 14). Previous data reported that normal glucose-tolerant patients with moderate to severe psoriasis were more insulin-resistant compared to controls suggesting that psoriasis may represent a pre-diabetic condition (15). It was also observed that homeostatic model assessment for insulin-resistance (HOMA-IR) values were higher in psoriatic patients compared to control subjects (16, 17) and that reduction of the Psoriasis Area and Severity Index (PASI) corresponded to a HOMA-IR values decrease (16). Insulin-resistance is also associated with inflammatory cytokines and adipocytokines secreted by adipocytes: levels were comparable between psoriatics and pre-diabetics (18). Furthermore, it was reported that in psoriatic patients, adiponectin (ADN) and visfatin concentrations were lower, while those of resistin and vaspin were higher with respect to values in control subjects, which suggests that their measurements could be useful to assess the psoriasis disease activity and prevent the onset of its comorbidities (19).

Endothelial damage markers such as soluble adhesion molecules [soluble intercellular adhesion molecule-1 (sICAM-1), soluble vascular adhesion molecule-1 (sVCAM-1), soluble E-selectin (sE-selectin)], and metalloproteinases [metalloproteinase-2 (MMP-2) and metalloproteinase-9 (MMP-9)] were found elevated in people with T2DM (20, 21) and also involved in psoriasis (22, 23). A previous report showed a rise of sICAM-1, sE-selectin, and MMP-9 levels and a trend toward the increase of sVCAM-1 concentrations in psoriatic patients with respect to controls. Since no correlation was found between PASI and these biomarkers, probably they represent an increased systemic rather than skin local inflammation (24).

High levels of plasminogen activator inhibitor-1 (PAI-1), a potent inhibitor of fibrinolysis, have been seen in patients with impaired glucose tolerance (IGT) (25), as well as insulin-resistance (26), and could play a role in the pathogenesis of psoriasis. One study showed that in pretreatment, PAI-1 plasma levels were increased in patients with psoriasis compared with healthy subjects, while during treatment they decreased, and this reduction correlated with PASI (27). Furthermore, it was reported that total PAI-1 levels were elevated in psoriatic patients, and they significantly decreased after treatment (24).

The aim of this study was to assess insulin-resistance, lipid abnormalities and cardiovascular risk biomarkers in psoriatic patients with or without T2DM.

## Methods and Materials

### Study Design

A case-control study was designed. All study participants provided written informed consent prior to enrollment. Patients were enrolled in the Department of Internal Medicine and Therapeutics, University of Pavia, and in the Institute of Dermatology, Fondazione IRCCS Policlinico, San Matteo, Pavia, Italy. The study protocol was approved by the local Ethical Committee (“Programma Ricerca corrente” clinical code 832-rcr2015-1) and registered (ClinicalTrials.gov NCT 02366272).

### Study Population

A total of 155 psoriatic patients (112M/43F) were enrolled in the study, subdivided into 86 patients with psoriasis only (59M/27F) (mean age 51.46 ± 15.87) and 69 with psoriasis plus T2DM (53M/16F) (mean age 62.67 ± 8.84). Diagnosis of plaque psoriasis was confirmed and assessed using PASI index by a dermatologist. Exclusion criteria for both groups were age <18 years old, previous phototherapy, and/or systemic therapy for psoriasis of at least 1-month duration, a diagnosis of erythrodermic or pustular psoriasis, pregnancy or breast-feeding. Furthermore, we also enrolled 120 T2DM (52M/68F) (mean age 60.98 ± 10.31) and 150 healthy subjects as a control population (82M/68F) (mean age 33.70 ± 8.40) to undergo the same testing.

### Assessments

Patients underwent initial screening assessment, including medical history, physical examination, evaluation of fasting plasma glucose (FPG), fasting plasma insulin (FPI), glycated hemoglobin (HbA_1c_), HOMA-IR, systolic blood pressure (SBP), diastolic blood pressure (DBP), total cholesterol (TC), low density lipoprotein-cholesterol (LDL-C), high density lipoprotein-cholesterol (HDL-C), triglycerides (Tg), lipoprotein(a) [Lp(a)], homocysteine (Hct), PAI-1, sICAM-1, sVCAM-1, sE-selectin, MMP-2, MMP-9, ADN, resistin, visfatin, and vaspin.

All parameters were determined after a 12 h overnight fast, determined 2 h after lunch. Venous blood samples were taken for all patients between 8:00 and 9:00 AM and were drawn from an antecubital vein with a 19-gauge needle, without venous stasis. We used plasma obtained by addition of Na2-EDTA, 1 mg/ml, and centrifuged at 3,000 × *g* for 15 min at 4°C. Immediately after centrifugation, the plasma samples were frozen and stored at −80°C for no more than 3 months. All measurements were performed in a central laboratory.

The Psoriasis Area and Severity Index was evaluated according to Fredriksson and Pettersson's rules (28). Psoriasis was defined as mild if PASI <10, moderate if between 10 and 20, severe if >20.

### Specific Assessment of Parameters

For a description of how various parameters were assessed see our previous papers (21, 29).

### Statistical Analysis

The Shapiro-Wilk test was used to test the normal distribution of quantitative variables. When quantitative variables were normally distributed, the results were expressed as the mean value and standard deviation (SD), otherwise the median and the interquartile range (IQR; 25–75th percentile) were reported. Qualitative variables were summarized as counts and percentages. One-way ANOVA or Kruskal-Wallis analysis of variance by ranks, as appropriate, with Bonferroni correction, was used to analyze differences among patient groups. Chi-square statistics or Fisher's exact test, as appropriate, was applied to compare qualitative variables. A *p* < 0.05 was considered statistically significant. All tests were two-sided. Data analysis was performed using the STATA statistical package (release 15.0 2018, Stata Corporation, College Station, Texas, USA).

## Results

### Study Sample

A total of 425 subjects were enrolled in the study (150 healthy controls, 120 diabetics, 86 psoriatics, and 69 diabetics with psoriasis). The characteristics of the study population are described in Table 1.

**Table 1:** Comparison of quantitative variables among the groups studied.

	**Controls**	**Diabetics**	**Psoriatics**	**Diabetics with psoriasis**
*N*	150	120	86	69
Sex (M/F) *n* (%)	82/68 (54.67/45.33)	52/68 (43.33/56.67)	59/27 (68.61/31.39)^°^^***^	53/16 (76.81/23.19)^*^
Age (years)	33.70 ± 8.40	60.98 ± 10.31^°°°°^	51.46 ± 15.87^°°°°^, ^****^	62.67 ± 8.84^°°°°^, ^∧^
Duration of DM (years)	0.00 [0.00–0.00]	6.00 [3.00–11.00]^°°°°^	0.00 [0.00–0.00]^****^	2.30 [1.75–2.60]^°°°°^, ^∧^
BMI (kg/m^2^)	23.18 ± 0.87	30.39 ± 4.92^°°°°^	27.14 ± 5.93^°°°°^, ^****^	29.04 ± 5.67^°°°°^, ^*^
BMI (18.5–24.9) *n* (%)	150 (100)	17 (14.17) ^°°°^	34 (40.00) ^°°°^, ^***^	9 (13.04) ^°°°^, ^*^
BMI (25–29.9) *n* (%)	0 (0.00)	41 (34.17) ^°°°^	32 (37.65) ^°°°^, ^***^	36 (52.17) ^°°°^
BMI (≥30) *n* (%)	0 (0.00)	62 (51.67) ^°°°^	20 (23.25) ^°°°^, ^***^	24 (34.78) ^°°°^, ^*^
FPG (mg/dl)	82.79 ± 13.29	133.30 ± 40.45^°°°°^	86.39 ± 8.04^****^	161.71 ± 71.14^°°°°^, ^****^, ^∧^
FPI (μU/ml)	6.80 [5.20–8.20]	12.35 [7.29–20.80]^°°°°^	9.70 [5.80–16.80]^°°°°^	25.80 [16.45–57.75]^°°°°^, ^***∧^
HbA_1c_ (%)	4.50 ± 0.53	6.99 ± 1.01^°°°°^	4.50 ± 0.53^****^	7.41 ± 1.36^°°°°^^∧^
HOMA-IR	1.40 ± 0.53	4.10 ± 2.64^°°°°^	2.07 ± 1.28^****^	10.26 ± 6.72^°°°°^^∧^
TC (mg/dl)	180.85 ± 25.80	168.31 ± 38.33°	199.27 ± 34.37^°°°°^, ^****^	204.29 ± 30.6^°°^, ^****^
LDL-C (mg/dl)	103.2 ± 12.7	92.2 ± 10.8°	115 ± 14.6^°°°°^, ^****^	118.8 ± 15.9^°°^, ^****^
HDL-C (mg/dl)	57.81 ± 12.50	48.98 ± 14.93^°°°°^	58.01 ± 15.13^****^	55.42 ± 13.11
Tg (mg/dl)	99.57 ± 41.70	139.88 ± 80.56^°°°°^	130.69 ± 71.88^°°^	151.75 ± 70.49^°°^
Lp(a) (mg/dl)	2.90 [2.40–7.20]	18.02 [4.32–46.00]^°°°°^	12.00 [6.00–23.00]^°°°°^	7.00 [4.50–30.50]^°°°°^
Hct (μmol/L)	7.74 ± 2.26	16.86 ± 6.34^°°°°^	23.78 ± 11.68^°°°°^, ^****^	24.83 ± 9.40^°°°°^, ^****^
PAI-1 (ng/ml)	15.24 [10.96–18.84]	47.97 [27.34–67.00]^°°°°^	24.1 [12.1–42.9]^°°^, ^**^	57.8 [20.54–74.96]^°°°°^, ^**^, ^∧^

*Data are represented as n and (%), means ± SD, median, and interquartile range [IQR]*.

° *p < 0.05 vs. controls*;

°° *p < 0.01 vs. controls*;

°°° *p < 0.001 vs. controls*;

°°°° *p < 0.0001 vs. controls*.

* *p < 0.05 vs. diabetics*;

** *p < 0.01 vs. diabetics*;

*** *p < 0.001 vs. diabetics*;

**** *p < 0.0001 vs. diabetics*.

∧ *p < 0.0001 vs. psoriatics*.

*DM, diabetes mellitus; BMI, body mass index; FPG, fasting plasma glucose; FPI, fasting plasma insulin; HbA_1c_, glycated hemoglobin; HOMA-IR, homeostasis model assessment insulin-resistance; TC, total cholesterol; LDL-C, low density lipoprotein-cholesterol; HDL-C, high density lipoprotein-cholesterol; Tg, triglycerides; Lp(a), lipoprotein(a); Hct, homocysteine; PAI-1, plasminogen activator inhibitor-1*.

### Body Mass Index

A significant BMI increase was observed in the studied groups (*p* < 0.0001 for all) compared with (Table 1). Lower BMI was obtained in psoriatics (*p* < 0.0001) and diabetics with psoriasis (*p* < 0.05) compared to diabetics (Table 1). Patients at normal weight were 100% in the control group while psoriatics and diabetics with psoriasis accounted for 14.17, 40.00, and 13.04%, respectively, of the diabetics group (*p* < 0.001 for all vs. controls; *p* < 0.001 for psoriatics and *p* < 0.05 for diabetics with psoriasis vs. diabetics) (Table 1). Overweight subjects were absent in the control group, whereas they made up 34.17% of the diabetics, 37.65% of the psoriatics, and 52.17% of the diabetics with psoriasis, respectively (*p* < 0.001 for all vs. controls and *p* < 0.001 for psoriatics vs. diabetics) (Table 1). Obese subjects were not found among the controls while they made up 51.67, 23.25, and 34.78% of the diabetics, psoriatics, and diabetics with psoriasis, respectively, (*p* < 0.001 for all vs. controls; *p* < 0.001 for psoriatics, and *p* < 0.05 for diabetics with psoriasis vs. diabetics) (Table 1).

### Glycemic Control

A significant FPG, HbA_1c_, FPI, and HOMA-IR increase was observed in diabetics and in diabetics with psoriasis (*p* < 0.0001 for all) compared to controls (Table 1). FPG, HbA_1c_, and HOMA-IR were found lower in psoriatics (*p* < 0.0001 for all) than in diabetics and higher in diabetics with psoriasis (*p* < 0.0001 for all) respect to psoriatics (Table 1). A significant FPG increase was observed in diabetics with psoriasis (*p* < 0.0001) compared with diabetics, whereas FPI levels were higher in psoriatics (*p* < 0.0001) than in controls and in diabetics with psoriasis compared with diabetics and psoriatics (*p* < 0.0001 for all) (Table 1).

### Lipid Profile and Lipoprotein Parameters

Total cholesterol and LDL-C levels were found lower in diabetics (*p* < 0.05) and higher both in psoriatics (*p* < 0.0001) and in diabetics with psoriasis (*p* < 0.001) compared with controls (**Table 1**). Also, psoriatics and diabetics with psoriasis showed high TC and LDL-C levels (*p* < 0.0001) compared to diabetics (Table 1). A significant decrease of HDL-C concentrations (*p* < 0.0001) was present in diabetics respect to controls whereas an increase was obtained in psoriatics (*p* < 0.0001) compared to diabetics (Table 1). Triglycerides and Lp(a) levels were higher in diabetics (*p* < 0.0001 for both), in psoriatics (*p* < 0.01 and *p* < 0.0001, respectively) and in diabetics with psoriasis (*p* <0.01 and *p* < 0.0001, respectively) compared to controls' baseline value (Table 1).

### Fibrinolytic Markers

A significant PAI-1 and Hct increase was recorded in diabetics (*p* < 0.0001 for both), psoriatics (*p* < 0.0001 and *p* < 0.01 respectively) and diabetics with psoriasis (*p* < 0.0001 for both) compared with controls (Table 1). Homocysteine levels were higher in psoriatics and diabetics with psoriasis (*p* < 0.0001 for both) compared with diabetics (Table 1). A significant decrease in PAI-1 values was observed in psoriatics (*p* < 0.01) compared with diabetics, whereas an increase was observed in diabetics with psoriasis compared with diabetics (*p* < 0.01) and psoriatics (*p* < 0.0001) (Table 1).

### Endothelial Parameters

A significant sICAM-1 and sVCAM-1 increase was observed in diabetics, psoriatics, and diabetics with psoriasis compared with controls (*p* < 0.0001 for both) while sE-selectin levels were higher only in diabetics with and without psoriasis (*p* < 0.0001) than in controls at baseline value (Table 2). A significant increase was obtained for sICAM-1 in diabetics with psoriasis compared with diabetics (*p* < 0.001) and psoriatics (*p* < 0.001) (Table 2). Levels of sVCAM-1 were lower in psoriatics (*p* < 0.0001) than in diabetics whereas they were higher in diabetics with psoriasis compared with diabetics (*p* < 0.05) and psoriatics (*p* < 0.0001) (Table 2). Moreover, a decrease of sE-selectin was obtained in psoriatics (*p* < 0.0001) with respect to diabetics whereas an increase was observed in diabetics with psoriasis (*p* < 0.0001) compared to psoriatics (Table 2).

**Table 2:** Comparison of soluble adhesion molecules, metalloproteinases, and adipocytokines among the groups studied.

	**Controls**	**Diabetics**	**Psoriatics**	**Diabetics with psoriasis**
*N*	150	120	86	69
sICAM-1 (ng/ml)	202.35 [175.68–248.63]	245.26 [207.42–301.29]^+^	254.64 [202.48–308.62]^+^	330.18 [273.86–407.66]^+^^,^ ^••^^,^ ^§§^
sVCAM-1 (ng/ml)	449.90 ± 127.99	627.50 ± 74.29^+^	545.13 ± 138.55^+^^,^ ^•••^	719.90 ± 172.37^+^^,^ ^•^^,^ ^§§§^
sE-selectin (ng/ml)	41.32 ± 16.02	60.03 ± 13.27^+^	40.93 ± 12.06^•••^	57.35 ± 19.97^+^^,^ ^§§§^
MMP-2 (ng/ml)	453.85 ± 105.84	752.94 ± 244.84^+^	615.40 ± 242.78^+^^,^ ^•••^	638.51 ± 289.37^+^
MMP-9 (ng/ml)	53.35 ± 16.24	65.03 ± 20.41^+^	75.27 ± 17.98^+^^,^ ^•••^	97.45 ± 15.17^+^^,^ ^•••^^,^ ^§§§^
ADN (μg/ml)	7.33 ± 0.45	4.28 ± 3.31^+^	3.52 ± 1.51^+^	2.09 ± 0.61^+^^,^ ^•••^^,^ ^§^
Resistin (ng/ml)	3.30 [3.10–3.60]	10.49 [9.25–12.59]^+^	4.82 [4.12–5.64]^+^^,^ ^•••^	5.10 [4.23–5.87]^+^^,^ ^•••^
Visfatin (ng/ml)	17.80 [16.20–23.80]	8.07 [7.30–9.00]^+^	5.86 [4.78–6.89]^+^^,^ ^•••^	5.88 [4.70–7.05]^+^^,^ ^••^
Vaspin (ng/ml)	0.53 [0.46–0.59]	1.54 [0.84–2.56]^+^	0.77 [0.57–1.32]^+^^,^ ^•••^	0.79 [0.62–1.30]^+^

*Data are means ± SD, median, and interquartile range [IQR]*.

+ *p < 0.0001 vs. controls*.

• *p < 0.01 vs. diabetics*;

•• *p < 0.001 vs. diabetics*;

••• *p < 0.0001 vs. diabetics*.

§ *p <0.05 vs. psoriatics*;

§§ p < 0.001 vs. psoriatics;

§§§ *p < 0.0001 vs. psoriatics*.

*sICAM-1, soluble intercellular adhesion molecule-1; sVCAM-1, soluble vascular cell adhesion molecule-1; sE-selectin, soluble E-selectin; MMP-2, metalloproteinase-2; MMP-9, metalloproteinase-9; ADN, adiponectin*.

Matrix metalloproteinase-2 and MMP-9 higher levels were observed in diabetics, psoriatics, and diabetics with psoriasis than in controls (*p* < 0.0001 for both) (Table 2). Moreover, MMP-2 levels were lower only in psoriatics (*p* < 0.0001) compared with diabetics (Table 2). A significant increase was observed for MMP-9 in psoriatics and diabetics with psoriasis (*p* < 0.0001) with respect to diabetics and in diabetics with psoriasis (*p* < 0.0001) compared with psoriatics (Table 2).

### Adipocytokines

Adiponectin and visfatin were lower, whereas resistin and vaspin were higher in diabetics, psoriatics and diabetics with psoriasis compared with controls (*p* < 0.0001 for all) (Table 2). A significant reduction was observed for ADN in diabetics with psoriasis compared with diabetics (*p* < 0.0001) and psoriatics (*p* < 0.05) (Table 2). Visfatin and resistin were decreased in psoriatics (*p* < 0.0001 for both) and in diabetics with psoriasis (*p* < 0.001 and *p* < 0.0001, respectively) while a significantly reduction of vaspin was observed only in psoriatics (*p* < 0.001) compared with diabetics (Table 2).

### PASI Subdivisions

Patient subdivisions according to PASI index were made, except for control and diabetic patients (Table 3). No significant differences were found among PASI groups (0 or absent psoriasis; < 10, or mild; 10–20, or moderate; >20, or severe) comparing psoriatic patients with patients with diabetes and psoriasis.

**Table 3:** PASI comparison among the groups studied.

	**Psoriatics**	**Diabetics with Psoriasis**
*N*	86	69
PASI (0 = absent) *n* (%)	4 (4.71)	0 (0.00)
PASI (< 10 = mild) *n* (%)	22 (25.58)	19 (27.53)
PASI (10–20 = moderate) *n* (%)	57 (67.06)	46 (66.66)
PASI (>20 = severe) *n* (%)	3 (3.53)	4 (5.79)

*Data are expressed as number (n) and percentage (%)*.

*PASI, Psoriasis Area and Severity Index*.

## Discussion

Psoriasis is a T cell-mediated inflammatory disease characterized by the expansion and activation of Th-1, Th-17, and Th-22 cells, which lead to local overproduction of multiple pro-inflammatory mediators both by lymphocytes and keratinocytes (10, 30, 31). Like psoriasis, T2DM is a complex disease with a multifactorial etiology, characterized by peripheral and hepatic insulin-resistance, as well as impaired insulin secretion from pancreatic beta cells. A pathogenetic link between psoriasis and T2DM could be traced primarily in the increase in psoriasis of TNF-α, a pro-inflammatory cytokine which plays an important role in the pathogenesis of insulin resistance, through the reduction of the tyrosine-kinase activity of the insulin receptor (14). Also, IL-23/Th17 axis was investigated, and its upregulation seems to be central in the explanation of cardiometabolic psoriasis comorbidity (4, 32).

As is well-evidenced by Davidovici et al. the production of pro-inflammatory molecules in psoriasis is not limited to the skin, and therefore psoriasis could be considered as a systemic disease (5, 10, 33, 34). The renowned concept of the “psoriatic march” suggests a growing and worsening systemic chronic inflammatory loop with pleiotropic effects on diverse processes, such as angiogenesis, insulin signaling, adipogenesis, lipid metabolism, and immune cell trafficking (33, 35, 36). Therefore, the metabolic aspects of psoriasis have the potential to impact other conditions such as obesity, diabetes, thrombosis, and atherosclerosis (10, 37).

Interestingly, our study shows that the levels of FPG, FPI, HbA_1c_, and the HOMA-IR are higher in psoriatics compared with controls, even if only FPI levels resulted statistically significant (*p* < 0.0001), supporting the hypothesis of psoriasis as a “pre-diabetic condition” (15). Moreover, biomarkers of insulin-resistance (FPG and FPI) were especially higher in diabetics with psoriasis compared with diabetics and controls, suggesting also a “positive” reinforcing action of psoriasis on diabetes. The absence of statistically significant differences in PASI subdivisions between psoriatic patients and diabetics with psoriasis, probably excludes similar reinforcing stimulus of diabetes on psoriasis. We could suggest that psoriasis worsens diabetes but that diabetes does not affect psoriasis, if we consider skin lesions. Diabetes, instead, contributes, together with psoriasis, to increase systemic cardiovascular risk. Psoriasis and diabetes together have more harmful action on metabolism than single diseases alone. In view of their lipid profile, psoriatics, and diabetics with psoriasis have TC and LDL-C values higher than controls, but also higher than diabetics. Boehncke et al. supports the synergic effect of the chronic state of inflammation caused by obesity, confirmed by our data where obesity was prevalent in diabetics and psoriatics (1, 10, 14, 38). Among other parameters, adipocytokines secreted by the adipose tissue show numerous functions including the metabolic regulation through lipid and glucose homeostasis. In particular, adiponectin is capable of increasing insulin sensitivity and has a vasoprotective effect acting as antioxidant and anti-inflammatory in the process of early atherogenesis. Our study shows significantly low levels of adiponectin in psoriatics compared to controls and in diabetics with psoriasis compared with diabetics, confirming that the association between psoriasis and diabetes worsens glycemic control and increases cardiovascular risk. Resistin could be considered as an adiponectin “antagonist;” it is higher in diabetics and to a lesser extent in psoriatics, without evidence of further increase in diabetics with psoriasis. Visfatin and vaspin do not yet have precise roles: probably, visfatin has an insulin-resistance activity, while vaspin is an insulin sensitizer. Various studies have reported obesity to be both a cause as well as consequence of psoriasis. In patients with obesity, a dysregulation of adipocytokines is known to occur with overproduction of pro-inflammatory adipocytokines such as leptin, visfatin, resistin, and interleukin-6 and decreased production of anti-inflammatory adipocytokines with insulin-sensitizing properties such as adiponectin and omentin. The soluble cells' adhesion molecules, such as sICAM-1, sVCAM-1, sE-selectin, and metalloproteinases MMP-2 and MMP-9, given the expression of endothelial dysfunction and plaque alteration, are increased in both diabetics and psoriatics compared with controls, with the exception of sE-selectin increased only in psoriatics compared with controls.

As far as fibrinolytic and vascular markers are concerned, PAI-1 plays a crucial role in the regulation of intravascular fibrinolysis, and its increase is associated with an increase in the risk of occurrence of thrombotic events. Homocysteine is instead a sulfur-containing amino acid, whose increase in the blood is associated with an increased risk of the onset of thrombotic events. Both PAI-1 and Hct are higher in diabetics than in controls and significantly higher in psoriatics than in controls; moreover, in diabetics with psoriasis there are also significantly higher PAI-1 and Hct values than in diabetics, strengthening the contention that psoriasis is associated *per se* with an increased cardiovascular risk (39–43).

A limitation of this study is that there is a significant difference in mean age between controls and other study groups: the lack of matching between case and control groups may interfere with the external validity of the study findings.

## Conclusion

In conclusion, with the limitations described above, this study suggested a close link between psoriasis and diabetes. Insulin resistance seems to be the key to understanding psoriasis comorbidities. Acknowledging psoriasis as a systemic disease, characterized by chronic inflammation, a widespread dysimmune background, and dysmetabolic surroundings, allows us to broaden the preventive and therapeutic approach to psoriasis. A healthy lifestyle, prevention and early diagnosis with appropriate therapies can be useful for both pathologies.

## Data Availability Statement

The original contributions presented in the study are included in the article/supplementary materials, further inquiries can be directed to the corresponding author/s.

## Ethics Statement

The studies involving human participants were reviewed and approved by IRCCS Policlinico San Matteo. The patients/participants provided their written informed consent to participate in this study.

## Author Contributions

GD, VBr, and PM designed the study, researched the data, and wrote the manuscript. CC and VBo researched the data and wrote the manuscript. AD'A processed and analyzed collected samples. All authors contributed to the article and approved the submitted version.

## Conflict of Interest

The authors declare that the research was conducted in the absence of any commercial or financial relationships that could be construed as a potential conflict of interest.
